# Effect of physical activity on the life quality of coronary artery bypass graft patients

**Published:** 2014-06-25

**Authors:** MG Firouzabadi, A Sherafat, M Vafaeenasab

**Affiliations:** Yazd Cardiovascular Research Center, Shahid Sadoughi University of Medical Sciences

**Keywords:** exercise, quality of life, SF=36, coronary artery

## Abstract

Abstract

Introduction. The quality of life depends on physical, psychological and social factors that are evidently influenced by the individual’s actions, prospect, attitude and behavior. Heart disease is one of the most imperative health problems in the world. Studies showed that exercise-based rehabilitation for patients with coronary artery disease effectively lowers the rate of cardiac death. The intent of this study was to determine the effects of physical activity on the life quality of cardiovascular patients after coronary artery bypass graft.

Materials and Methods. This randomized clinical trial was performed on two groups of coronary artery patients of Yazd Afshar hospital. All the 70 participants were post surgery coronary artery patients who were divided into two groups. Data was collected by two questionnaires: A personal information questionnaire and a quality of life questionnaire (SF=36). Data of both groups was collected in the first and fourth month after the discharge from surgery and sessions were analyzed by SPSS 16 and by using T-test and Chi-square.

Results. Results displayed that after the intervention, all the categories of the components of the quality of life were increased except for general health. There was no significant differentiation between these statuses in the control group and the premier grades about the components of the social function (88.98 out of 100). The comparison of total scores of the quality of life before the intervention showed the quality of life of both groups one month after surgery was not significant (p=75%), but in four months after surgery, the distinction between the mean grade scores of the intervention, the group was considerable (p=0.0001).

Conclusion. The comparison of the total scores of the quality of life indicated an increase in the scores of the quality of life in the intervention group after the exercise.

## Introduction

Quality of life in relation to health is defined by the individual’s subjective evaluation of the current health, health care and health promotion activities [**1**]. The quality of life depends on physical, psychological and social factors that are evidently influenced by the individual’s actions, prospect, attitude and behavior [**2**]. Quality of life is an amalgamation of 9 indicators: 1- leisure and culture, 2- cost of living, 3- risk and safety, 4- communication, 5-health, 6- liberty, 7- ecology, 8- economy and 9- climate. Enhancing the quality of life is one of the main objectives in the treatment of heart diseases. 

 Heart disease is one of the most imperative health problems in the world. The escalating morbidity rate and disability caused due to heart disease in adults is followed by social damage, and though scientific advances and cardiovascular surgery improved the patient’s life conditions [**3,4**], nevertheless, some studies divulge that life quality improvements of postoperative patients was poor [**5,6**] and individuals suffering from coronary artery disease, tend to experience changes in morals and values as opposed to before the diseased condition. Activity limitations and the everyday state of affairs of patients with coronary artery disease have adverse effects on their lifestyle [**7**]. 

 The cardiac rehabilitation program is a comprehensive program including physical activity, exercise, nutrition and psychological counseling, blood pressure, blood lipid and blood glucose control, smoking cessation. 

 This Program is one of the ways recommended for the rehabilitation and improvement of the quality of life of patients after open-heart surgery and also the prevention of future complications. 

 Studies showed that exercise-based rehabilitation for patients with coronary artery disease effectively lowers the rate of cardiac death [**3**]. Findings from a review of 22 randomized clinical trials of exercise after myocardial infarction showed that exercise decreases the possibility of re-infarction and the risk of cardiovascular mortality [**4**]. 

 The results of two systematic reviews including 48 randomized controlled trials revealed a 20% reduction in mortality and 27% decrease in cardiovascular mortality rate in the second to fifth year of the diseased condition [**7,8**]. 

 Although cardiac rehabilitation is an imperative component of care for cardiac patients [**9**], however, the amount of people involved and participating in rehabilitation for these patients are low, even in developed countries [10,11]. In our country, there is no ongoing rehabilitation after hospital discharge. Unfortunately, the rate of rehabilitation referrals assigned by doctors or hospitals is exceptionally nominal [**12**]. 

 Increasing rates of cardiovascular diseases and the immense differentiation between the number of patients in comparison with other countries also rising numbers of the lower aged (according to global statistics) patients in need of surgery, are the importance of the study of the quality of life after surgery. Because of the importance of improving the quality of life for the prevention of fatality in cardiac rehabilitation patients, attention to the quality of life and the role of rehabilitation is much required. The intent of this study was to determine the effects of physical activity on the quality of life of cardiovascular patients after coronary artery bypass graft.

## Materials and Methods

This randomized clinical trial was performed on two groups of coronary artery patients of Yazd Afshar hospital. All the 70 participants were post surgery coronary artery patients who were divided into two groups (35 in the intervention group and 35 in the nonintervention group). Data was collected by two questionnaires: A personal information questionnaire and a quality of life questionnaire (SF=36), its reliability and validity being confirmed in numerous studies [**2,6**]. 

 SF-36 questionnaire consists of 36 questions, all with 8 components. The questions are of two, three, five and six choices. The questions for the physical function are 10, physical role limitation 4, body pain 2, general health 6, vital force 4, social function 2, psychological role limitation 3 and mental health 5 [**15**]. The range of score was of 0-100. Participants of this study were selected from patients discharged from the hospital’s surgery wing using random numbers table. They were divided into two groups and the data were collected in the month following discharge. 

 The intervention for the intervention group included: 24-32 sessions of physical activity, three times a week for one to one and a half hours respectively. The exercise sessions were under supervision of medical and CCU nurses, and based on the status of the patients, included 20 minutes warm-up, 20-40 minutes aerobic exercise (such as bikes and treadmills), 5 minutes cooling and 20 minutes relaxation. No intervention was taken into account for the control group. At the end of four months from discharge and the physical activity intervention, the SF-36 quality of life questionnaire was completed for both groups for a second round.

 Data of both groups that was collected in one and four months after discharge from surgery and sessions were analyzed by SPSS 16 and by using T-test and Chi- square.

## Results

The mean ages for intervention and control groups were 59.17±8.48 and 58.85±8.26 years respectively. Both groups were similar in age, gender and marital status. About 40% of the control group and 37.1% of the intervention group had a primary school graduation. There was no significant differentiation in the education levels of the two groups. 28.6% of the participants were housewives and 25.7% of them were retired. Also, there was no significant dissimilarity between the two groups job status.

 Results displayed that after the intervention, all the categories of the components of quality of life were increased except for general health. There was no significant differentiation between these statuses in the control group and the premier grades were about the components of the social function (88.98 out of 100). The lowest grade was about the mental health (45 out of 100). Paired T-test showed a considerable difference between the mean grade scores of all components of the quality of life before and after physical activity in the intervention group with p=0.0001, but no significant difference between the mean grade scores of seven components of the quality of life of the control group, in one and four months after discharge from surgery sessions (**Table 1**). 

 The comparison of total scores of quality of life before the intervention showed the quality of life of both groups one month after surgery was not significant (p=75%), but in four months after surgery the distinction between the mean grade scores of the intervention group was considerable (p=0.0001) (**Table 1**).

**Table 1:**
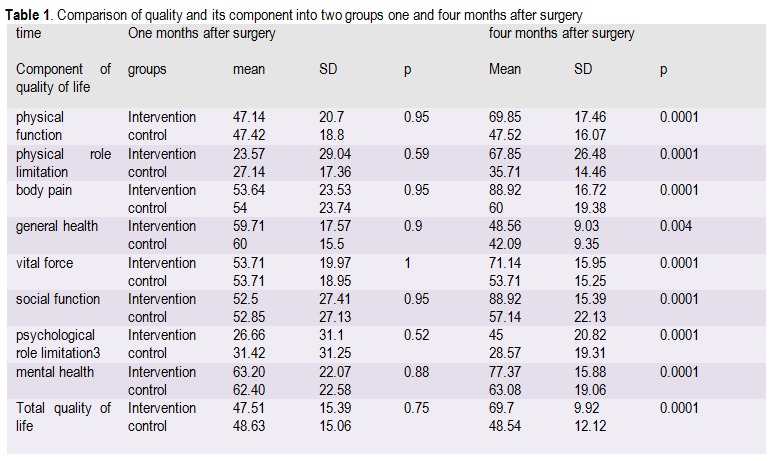
Comparison of quality and its component into two groups one and four months after surgery

## Conclusion

Results show that although the mean scores of physical functioning of both two groups before intervention was unsubstantial, after the intervention (physical activity in the intervention group), the scores compared with the control group increased significantly. These results were the same as the ones of Brown [**14**], Berobiker [**15**], Hadian [**16**]; in all of these studies the physical function of the quality of life being augmented. 

 The results in the physical role limitation showed that the mean grade scores in this component was similar in both the intervention and control groups before the exercise, but improved considerably after the intervention (p=0.0001). Our conclusions were the same as the results of Berobiker [**15**], but were different from the results of Brown [**14**]. Also, some studies in Iran have reached these same results [**17-19**].

 Our data revealed that the mean grade scores of physical pain that were not significantly different before intervention, had considerable scores after the intervention (p=0.0001). These results were not the same as the results of Brown’s study [**14**], but were conclusive with results of many studies that took place in Iran and other countries [**16,18,20**].

 General health scores four months after the surgery were significantly reduced in both groups (p=0.0001). These findings were different from all the other results [**13-15,17,18,20**]. This reduction in the scores may possibly be due to the fact that in the SF-36 questionnaire, the component of general health is dependent on the respondent’s feeling towards themselves and may differ at different times. 

 The results in the vital force component showed that the mean grade scores in this component was similar both in the intervention and control groups before exercise, but the mean grade score increased significantly in the intervention group after the intervention (p=0.0001). Our results were the same as the Berobiker’s results [**15**], but different from the results of Brown [**14**]. Some studies in Iran reached the same results [**16,18**].

 Social functioning data revealed that the mean grade scores of this component that were not significantly different before the intervention, had significant scores afterwards (p=0.0001). These results were not the same as the results of the Brown’s study [**17**], but were the same as the results of many studies done in Iran and other countries [**15,17,20**].

 An evaluation of the mean grade scores of psychological role limitation showed significantly higher scores in the intervention group as opposed to the control group after exercise. Based on Brown’s results, physical activity and rehabilitation do not affect the psychological role limitation [**14**], but the results of Chakraborty were the same as our results [**20**]. The results of our study in this component were also the same as the results of Berobiker [**15**] and other studies [**18,19**]. 

 An assessment of the mental health scores in the two groups showed that physical activity increased this score in the intervention group. The results of numerous other studies were the same as our results [**18,20**], with the exception of the results of Brown [**14**]. 

 The comparison of the total scores of the quality of life indicated an increase in the scores of the quality of life in the intervention group after exercise. 

 Limitation

 The major limitation of this study was the unwillingness of the patients to participate in the study.
